# The spillover effect of midwife attrition from the Nigerian midwives service scheme

**DOI:** 10.1186/s12913-018-3106-x

**Published:** 2018-04-23

**Authors:** Daniel O. Erim, Harrison E. Offiong, Christine Kim, Folasade A. Bello, Jeremy Moulton, Stephanie B. Wheeler, Harsha Thirumurthy

**Affiliations:** 1 Department of Health Policy and Management, the University of North Carolina at Chapel Hill, Chapel Hill, NC 27599 USA; 2 University Hospitals of Leicester NHS Trust, Leicester, UK; 3 Department of Obstetrics and Gynecology, University of Ibadan, Ibadan, Nigeria; 4 Department of Public Policy, University of North Carolina at Chapel Hill, Chapel Hill, USA; 5 Department of Medical Ethics and Health Policy, University of Pennsylvania, Philadelphia, USA

**Keywords:** Midwives service scheme, Maternal mortality in Nigeria, Maternal policy intervention, Midwife attrition, Policy evaluation

## Abstract

**Background:**

The Nigerian Midwives Service Scheme (MSS) increased use of antenatal services at rural public sector clinics. However, it is unclear if women who would not have otherwise sought care, or those who would have sought care in rural private sector clinics caused this change. Additionally, it is also unclear if the reported midwife attrition was associated with a spillover of the scheme’s effect on urban areas. We sought to answer these two questions using data from two nationally representative surveys.

**Methods:**

We used an interrupted time series model to assess trends in the use of obstetric (i.e. antenatal and delivery) services among rural and urban respondents in the 2008 and 2013 Nigerian demographic and health surveys.

**Results:**

We found that the MSS led to a 5-percentage point increase in the use of antenatal services at rural public sector clinics, corroborating findings from a previous study. This change was driven by women who would not have sought care otherwise. We also found that there was a 4-percentage point increase in the use of delivery services at urban public sector clinics, and a concurrent 4-percentage point decrease in urban home deliveries. These changes are most likely explained by midwives’ attrition and exemplify a spillover of the scheme’s effect.

**Conclusion:**

Midwife attrition from the Nigerian MSS was associated with a spillover of the scheme’s effect on the use of delivery services, on urban areas.

## Key messages

Midwife attrition from the Nigerian Midwives Service Scheme was associated with greater use of delivery services in urban areas (a spillover effect).

## Background

In December 2009, the Nigerian government introduced the *Midwives Service Scheme* (MSS) to provide greater access to obstetric services in rural areas. [1–3] The scheme was a policy response to the high maternal mortality ratio. [4, 5] About 2500 midwives were mobilized (from among unemployed midwives [45%], new graduates [44%] and retirees [11%]) and deployed to 652 upgraded rural public sector clinics in all parts of the country (more to the North than the South) in July 2010. [1–4] This mobilization and deployment may have increased the pre-existing national and rural midwives’ workforce by about 3% and 9% respectively (see Table 1), and each clinic was supposed to receive four midwives who would work in shifts to provide 24-h coverage. [2, 6] Despite its large scope, the scheme had a limited impact on use of obstetric services. [1–3] For instance, Okeke et al. (2016) showed that in its first year, the scheme increased use of antenatal services at rural public-sector clinics by 5-percentage points, but had no effect on the use of delivery services at these clinics. [2, 3] However, little is known about whether these changes were driven by women who would not have sought antenatal care otherwise, or by women who moved from private sector clinics (which have the lowest number of midwives per capita [see Table 1] but may offer higher quality care at higher out of pocket costs]), drawn by the potentially lower healthcare cost. [7] This difference is important because women in the former group were the scheme’s intended beneficiaries, and are more likely to affect maternal health indicators than those in the latter. Additionally, for various reported reasons (including irregular remuneration and unavailability of secure/decent housing), midwives started deserting their postings for more lucrative and secure urban practices in late 2010. [1–3, 8–11] While the pattern of attrition is unclear, one study suggests that by 2003, 1 in 5 deployed midwives (up to half or all in some Northern states) left the scheme. [8] However, little is known about whether midwife attrition was associated with changes in use of obstetric services in urban areas (i.e. a spillover effect). [12–14] Such information is useful to evaluators and relevant stakeholders of the scheme. We sought to answer these two questions using data from two nationally representative surveys.

**Table 1:** A selection of demographic indices and measures of obstetric care capacity

Demographic indices	National	Northern zones	Southern zones
Population size (2006) [35]	140	71	69
Number of women aged 15 – 49 years (million) [17, 35]	35	20	15
Number of women aged 15 – 49 years in rural areas (million) [17, 35]	21	15	6
Number of women aged 15 – 49 years in urban areas (million) [17, 35]	14	5	9
Prevalence of pregnancy in women aged 15 – 49 years (%) [17]	12	14	8
Distribution of midwives before the Midwives Service Scheme			
Total number of midwives [36, 37] ^a, b^	89,000	37,000	52,000
Number of midwives in rural areas [38] ^a, b, c^	30,000	12,000	18,000
Number of midwives in urban areas [38] ^a, b, c^	59,000	25,000	34,000
Number of midwives per 100,000 population [36] ^b^	68	52	75
Nurses & midwives per 100,000 population in rural public sector clinics [39]	64	–	–
Nurses & midwives per 100,000 population in rural private sector clinics [39]	24	–	–
Nurses & midwives per 100,000 population in urban public sector clinics [39]	121	–	–
Nurses & midwives per 100,000 population in urban private sector clinics [39]	63	–	–
Annual number of graduating nurses and midwives [40]	5500	NA	NA
Some measures of primary care clinics’ capacity (rural and urban) [12]			
Can always provide emergency obstetric care (%)	41	–	–
Can administer injectable antibiotics (%)	79	–	–
Can diagnose and treat eclampsia (%)	23	–	–
Can conduct assisted vaginal delivery (%)	10	–	–
Can diagnose and treat severe shock (%)	36	–	–
Can safely transfuse blood (%)	20	–	–
Can conduct Caesarian section (%)	12	–	–
Has at least one doctor that is constantly available (%)	20	–	–
Has at least one nurse/midwife that is constantly available (%)	48	–	–
Has at least one obstetrician that is constantly available (%)	8	–	–
Has at least one anesthesiologist that is constantly available (%)	7	–	–
Has an ambulance for transporting pregnant women to referral clinics (%)	5	–	–
Has at least one labor ward (%)	79	–	–
Has at least one delivery room (%)	74	–	–
Has at least one functional operating room (%)	16	–	–
Receives uninterrupted electricity supply whenever there are obstetric emergencies (%)	15	–	–

NA, not applicable

a – Estimates are rounded to the nearest thousand

b – The zonal distribution (or densities) of midwives are plausible estimates

c – The national distribution of midwives are plausible estimates

### Overview

Details about the MSS are described elsewhere. [1–3] In brief, the program’s goal was to drive down maternal mortality by increasing the use of obstetric services at selected rural public sector clinics. [1] This goal was to be achieved by both demand-side interventions (e.g. health education campaigns and advocacy by influential individuals in the beneficiary communities), [1–3] and supply-side interventions (e.g. mobilizing and deploying midwives to 652 upgraded rural public sector clinics, serving about half of the population in need). [4, 5] A complementary policy intervention called SURE-P MCH (Subsidy Reinvestment and Empowerment Program for Maternal and Child Health) was introduced in 2012 to cover the remaining half of the population in need. [15]

## Methods

### The data

We used data from the 2008 and 2013 Nigerian Demographic and Health Surveys (NDHS). [16, 17] Survey respondents (women aged 15 – 49 years) were randomly selected by probability sampling. [18] The datasets contain information on respondents’ socio-demographic characteristics (e.g. age, level of education and rural/urban residence), and use of obstetric services going back six years (i.e. 2003 – 2008 and 2008 – 2013 in the 2008 and 2013 DHS respectively). [18] We combined both 2008 and 2013 datasets to have adequate pre- and post-implementation data, and focused on the most recent birth to minimize recall bias. We also excluded respondents without a birthing experience and those that delivered in 2012 and 2013 (because of SURE-P MCH).

### Identification strategy

DHS staff designated respondents’ residences as rural or urban. Identifying DHS questions and their responses are presented in Table 2. Eligible respondents reported which obstetric (i.e. antenatal and delivery) service they used during their most recent pregnancy. We used these data to create our binary outcome variables, which are as follows:

1. Use of antenatal services:
  - one or more antenatal visits to public-sector clinics;
  - one or more antenatal visits to private-sector clinics;
  - non-use of antenatal services (including those who reported “home” or “other” as sites of antenatal care)
2. Use of delivery services:
  - Delivered at a public-sector clinic;
  - Delivered at a private-sector clinic;
  - Delivered at home/other sites.

**Table 2:** Relevant DHS questions used in this analysis

	DHS questions	Responses
1	Where did you receive antenatal care for this pregnancy?	Home − your home − other home Public sector − Government hospital − Government health center − Government health post/dispensary − Other public sector (site) Private med. Sector − Private hospital/clinic − Other private med. Sector Other
2.	How many times did you receive antenatal care during this pregnancy?	Number of times/Don’t know
3.	Where did you give birth?	Home − your home − other home Public sector − Government hospital − Government health center − Government health post/dispensary − Other public sector site Private med. Sector − Private hospital/clinic − Other private med. Sector Other
4.	Many different factors can prevent women from getting medical advice or treatment for themselves. When you are sick and want to get medical advice or treatment, is each of the following a big problem or not?	
	• Getting permission to go to the doctor?	Big problem or not a big problem
	• Getting money needed for advice or treatment?	Big problem or not a big problem
	• The distance to the health facility?	Big problem or not a big problem
	• Not wanting to go alone?	Big problem or not a big problem
	• Attitude of the health workers?	Big problem or not a big problem

Thus, we had six outcome variables, which allowed us to keep track of the “origin” of those who drove noticeable changes in obstetric care utilization trends.

### Analytic approach

We made the following assumptions: that there was no other large-scale intervention to increase demand for obstetric services; [1–3] that selected public sector clinics were in rural communities with severely limited access to obstetric care; [1–3, 9] and that pregnant women seek obstetric care at sites close to where they reside. [12–14, 19] We used an *interrupted time series* (ITS) model to specify trends and assess for changes in use of obstetric services during the pre- and post-implementation periods (i.e. 2003 – 2009 and 2010 – 2011 respectively). This model relies on the assumption that respondents did not select into either of these periods. [2, 3] It also allows us to “sort” eligible respondents by their delivery year, which makes trends in use of obstetric services more obvious. Our first step was to use the ITS model to assess trends in use of obstetric services among rural respondents. We did this to validate our approach, and to see where those who drove the increase in use of antenatal services were coming from (i.e. private sector clinics or non-use/home). [2, 3] Next, we used the ITS model to assess trends in use of obstetric services among urban respondents. Under the null hypothesis, there should be no changes. Our model specification is as follows:

*Y = α + β*_*1*_*(Year*_*i*_ *− 2010) + β*_2_*(Year_*i*_ ≥ 2010)*(Year_*i*_ *− 2010) + β*_3_*(Year_*i*_ *≥ 2010) + X*_*i*_ *+ ε*_*i*_*.*

*Y* is the outcome variable, and represents any of the six binary indicators of use of obstetric services (1a – 1c and 2a – 2c above) for each respondent. *α* is the constant term, and *Year*_*i*_ represents the year of most recent birth, re-centered around year 2010 for each respondent. The coefficient of this re-centered variable (*β*_*1*_) captures the indicated trend in use of obstetric services. We included this re-centered variable interacted with an indicator of the pre- and post-implementation periods. Its coefficient (*β*_*2*_) represents the difference between the pre- and post-implementation trend in use of obstetric services. *β*_*3*_ measures the size of the vertical distance between both pre- and post-implementation trends in 2010 and thus estimates the program’s *intention-to-treat* effect (ITTE, for rural respondents) and spillover effect (for urban respondents). Our control variables included respondents’ years of formal education, wealth quintile, reported barriers to accessing care (see Table 2 – item 4, all captured as binary variables), and state fixed-effects (to minimize bias from state-level variation in government subsidies for usual out-of-pocket obstetric care costs [at select public-sector clinics] on access to obstetric care). *ε*_*i*_ represents the error term. We used survey sampling weights in our regression models, and clustered observations by geopolitical zones (informed by variations in the allocation of MSS resources). [1, 2] As in Okeke et al.’s [2016] study, [2, 3] we conducted national- and regional-level (Northern and Southern regions) analyses. We conducted regional-level analyses because we were concerned about potentially larger effect sizes in the North (it had more deployed midwives and MSS clinics and a higher attrition rate than in the South) or in the south (due to the likelihood for greater uptake of obstetric care services than in the North). [16, 17, 20, 21]

## Results

### Characteristics of the sample

Respondents’ characteristics are presented in Table 3. Urban respondents were about a third of the sample. Compared to rural respondents, urban respondents a) had more years of formal education, b) had fewer children, c) were more likely to use obstetric care services d) were less likely to be in the middle or a lower wealth quintile, and e) were less likely to report barriers to accessing primary care. The table also contains the size and significance of unadjusted differences in the means of relevant individual-level characteristics between rural and urban respondents’ pre- and post-implementation groups. Regional level characteristics are not presented.

**Table 3:** A comparison of dependent and independent variables for rural and urban respondents in the pre- and post-intervention groups

	Rural respondents				Urban respondents			
Characteristics	Last childbirth occurred before 2010	Last childbirth occurred between 2010 - 2011	Difference	T-test *p*-value	Last childbirth occurred before 2010	Last childbirth occurred between 2010 - 2011	Difference	T-test *p*-value
Mean age (years)	29.9	28.5	−1.4	< 0.001	30.9	29.7	−1.2	< 0.001
Average number of children ever born	4.4	4.2	− 0.2	< 0.001	3.9	3.6	−0.3	< 0.001
Mean number of years of formal education received	3.4	3.4	0.0	< 0.956	7.4	8.1	+ 0.7	< 0.001
Percentage in the middle or a lower wealth quintile	83.3	84.0	0.7	0.128	28.4	27.9	−0.5	0.532
Other characteristics								
Any antenatal visit to public-sector clinics (%)	36.1	45.5	+ 9.4	< 0.001	54.4	59.6	+ 5.2	< 0.001
Any antenatal visit to private clinics (%)	9.2	7.6	−1.6	< 0.001	26.6	26.6	0.0	0.982
Antenatal visits to other sites/No antenatal care	52.8	46.3	−6.5	< 0.001	18.3	13.4	−4.9	< 0.001
Deliveries in public-sector clinics/hospitals (%)	16.3	18.0	+ 1.7	< 0.001	34.9	39.1	+ 4.3%	< 0.001
Deliveries in private-sector clinics/hospitals (%)	7.9	6.3	−0.16	< 0.001	24.4	25.6	+ 1.1	0.162
Deliveries at home (%)	74.7	75.4	+ 0.7	0.208	37.9	35.0	−2.9	0.001
Barriers to accessing primary healthcare services								
-those in need of permission (%)	16.4	14.7	−1.7	< 0.001	11.4	6.7	−4.7	< 0.001
-those who couldn’t afford the cost of care (%)	63.3	52.1	−11.2	< 0.001	43.1	33.1	−10.0	< 0.001
-those who thought the closest clinic/hospital was too far (%)	46.2	40.0	−6.3	< 0.001	20.5	15.7	−4.8	< 0.001
-those in need of company (%)	21.2	18.1	−3.1	< 0.001	9.6	7.0	−2.6	< 0.001
Number of observations	15,495	11,806	–	–	6224	5737	–	–

The pre- and post-intervention groups made up of respondents whose most-recent birth occurred between 2003 and 2009 and 2010 – 2013 respectively. In the difference column, “+” indicates an increase, while “-” indicates a decrease. The significant reduction in barriers to accessing primary healthcare services over time may be a consequence of the MSS, or may mediate the effect of the scheme. However, we chose to leave them in the models to avoid *omitted variable bias,* and because our conclusions were robust to their exclusion

### Effect on rural respondents

**Fig. 1:**
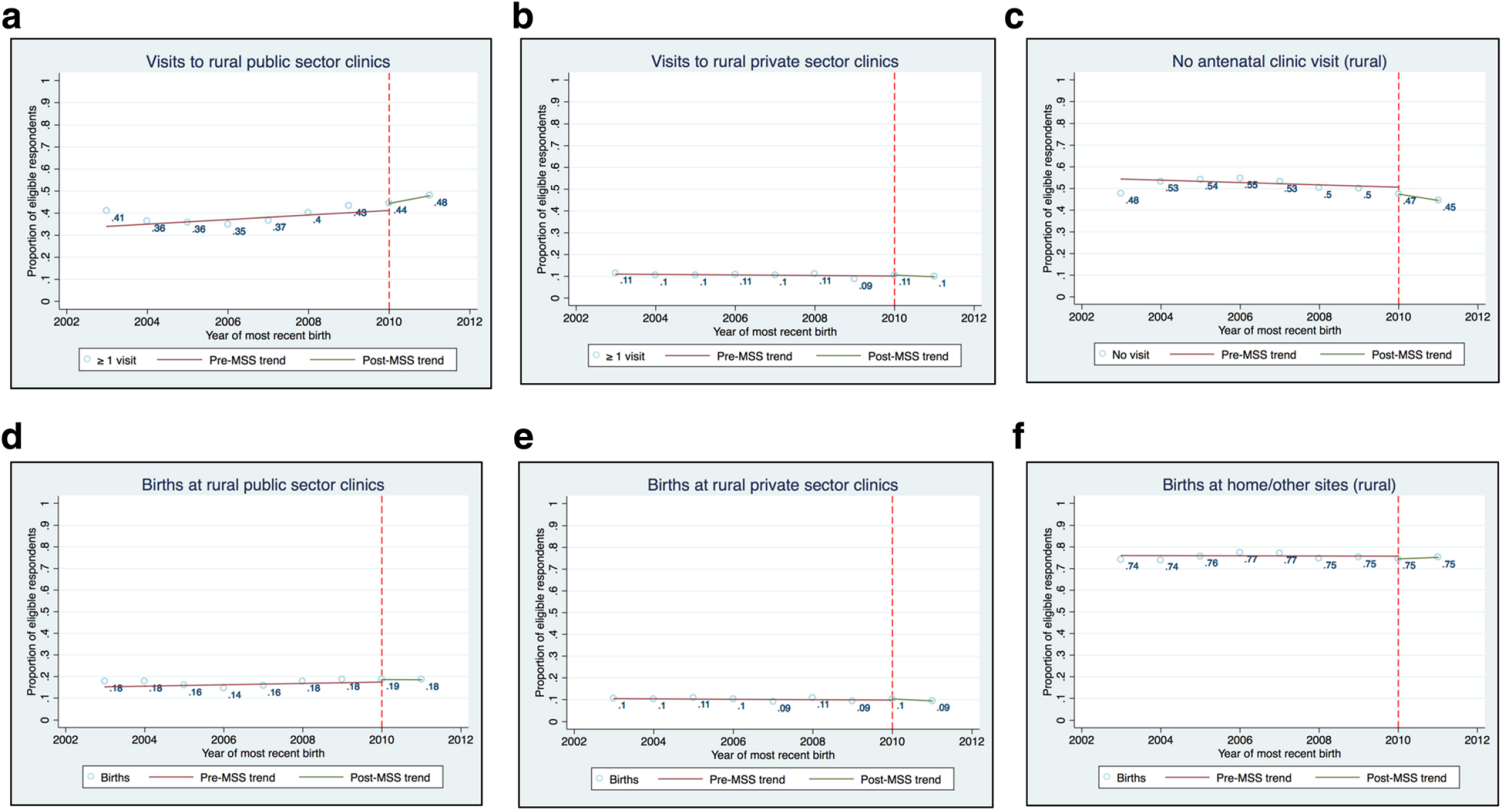
Changes in obstetric care utilization in rural areas

Our estimates of the scheme’s ITTE are presented in Table 4. At the national level, the MSS increased antenatal visits to public-sector clinics by 5 percentage points (*p*-value = 0.001) in 2010. This change was accompanied by a 5 percentage points decline (p-value = 0.006) in non-use of antenatal care services in the same year. We found no evidence of any change in use of antenatal services at private-sector clinics. We also found no evidence of any change in the use of delivery services. Trends in the use of obstetric services among rural respondents are presented in Fig. 1.

**Table 4:** The estimated intention to treat effect (ITTE) of the MSS on rural respondents

Outcomes	Antenatal visits			Delivery sites		
	Public sector clinics	Private sector clinics	No antenatal care	Public sector clinics	Private sector clinics	Home
National						
Effect in 2010	0.050***	−0.002	− 0.051***	0.016	0.002	− 0.013
	(0.016)	(0.006)	(0.011)	(0.011)	(0.008)	(0.008)
Pre-MSS trend	0.001	−0.001	−0.003	0.002	−0.001	−0.001
	(0.003)	(0.002)	(0.007)	(0.002)	(0.001)	(0.004)
Observations	25,562	25,065	26,059	26,328	26,401	26,401
R-squared	0.211	0.211	0.338	0.195	0.216	0.376
Northern zones						
Effect in 2010	0.053***	0.003	−0.056**	0.010	0.008	−0.016
	(0.003)	(0.003)	(0.007)	(0.006)	(0.007)	(0.014)
Pre-MSS trend	0.009	0.0008	−0.009	0.006*	−0.001	− 0.006
	(0.011)	(0.001)	(0.009)	(0.002)	(0.001)	(0.002)
Observations	19,100	19,100	19,474	19,777	19,777	19,777
R-squared	0.251	0.156	0.316	0.161	0.158	0.273
Southern zones						
Effect in 2010	0.039	−0.018	−0.026	0.034	−0.021	0.004
	(0.045)	(0.017)	(0.035)	(0.030)	(0.020)	(0.002)
Pre-MSS trend	−0.004	−0.002	0.011	−0.008	−0.001	0.011*
	(0.007)	(0.007)	(0.022)	(0.006)	(0.005)	(0.004)
Observations	5965	5965	6585	6624	6624	6624
R-squared	0.127	0.164	0.244	0.124	0.159	0.289

Robust standard errors are in parentheses. *** *p* < 0.01, ** *p* < 0.05, * *p* < 0.1 Pre-MSS trend, pre-implementation trend (i.e. the trend in the indicated outcome prior to implementation of the midwives service scheme)

In the Northern region, there was a 5.3 percentage point increase (*p*-value = 0.003) in antenatal visits to public sector clinics. This change was also accompanied by a 5.6 percentage point decline (p-value = 0.016) in non-use of antenatal care services in the same year. We found no evidence of any change in use of obstetric care services at Northern private-sector clinics. We also found no evidence of any change in the use of obstetric care services in the southern zones.

### Spillover effects

**Fig. 2:**
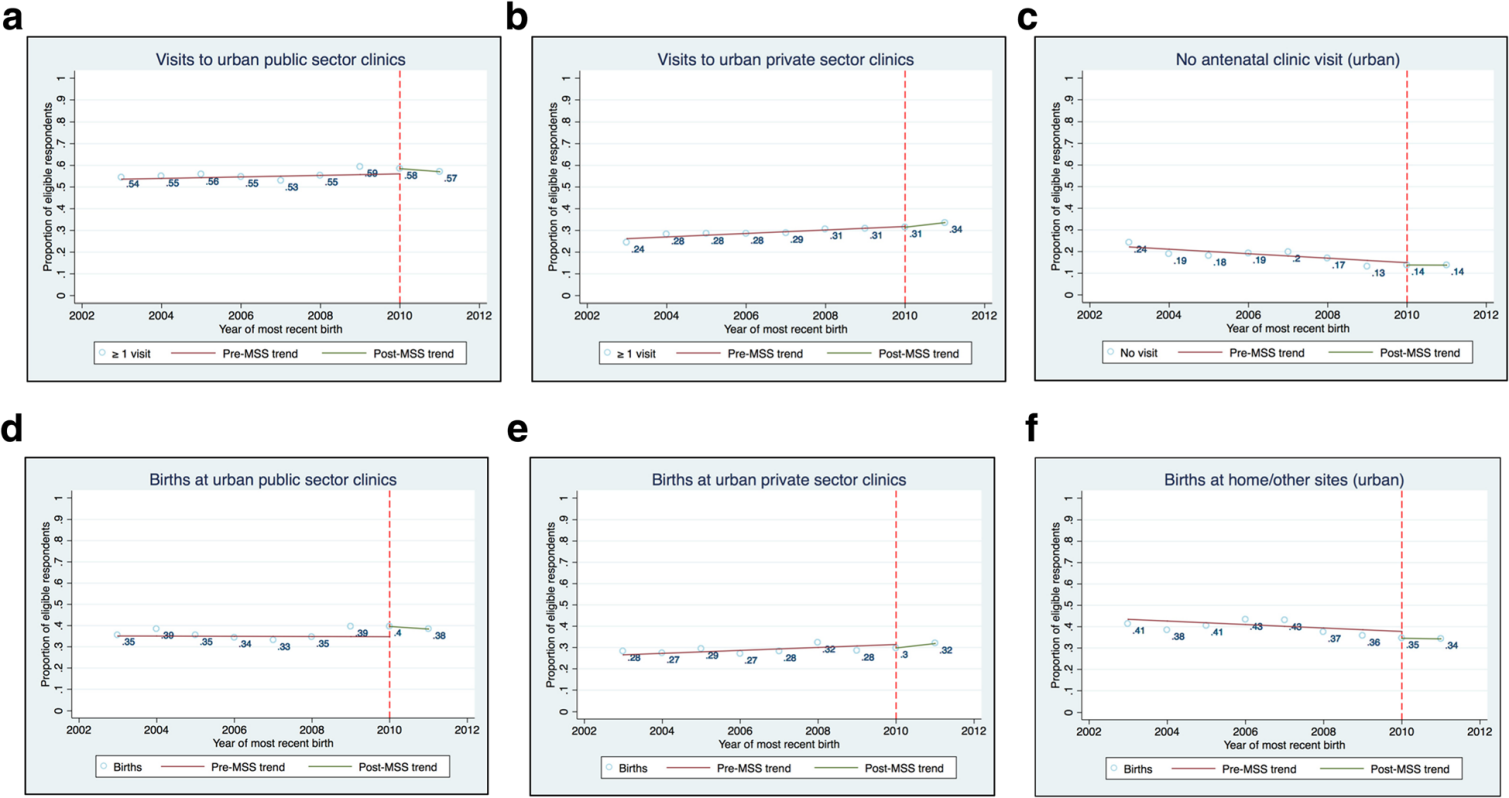
Changes in obstetric care utilization in urban areas

Our estimates of the program’s spillover effects are presented in Table 5. At the national level, we found no evidence of any change in use of antenatal services at urban public sector clinics, and a 4 percentage points increase (*p*-value = 0.041) in the use of delivery services at urban public sector clinics. We also found no evidence of any change in the use of obstetric services at urban private sector clinics. Trends in the use of obstetric services among urban respondents are presented in Fig. 2.

**Table 5:** The estimated “spillover” effect of the MSS on urban respondents

Outcomes	Antenatal visits			Delivery sites		
	Public sector clinics	Private sector clinics	Other sites/no antenatal care	Public sector clinics	Private sector clinics	Other sites/home
Spillover effect	0.017	−0.004	−0.011	0.041**	−0.009	− 0.020
	(0.026)	(0.015)	(0.011)	(0.015)	(0.010)	(0.014)
Pre-MSS trend	0.003	0.001	−0.004	−0.003	0.004	0.004
	(0.006)	(0.005)	(0.004)	(0.005)	(0.004)	(0.006)
Observations	10,660	10,660	11,374	11,516	11,516	11,516
R-squared	0.158	0.250	0.197	0.120	0.246	0.362
Northern region						
Spillover effect	0.027	−0.019	−0.003	0.054**	−0.015	− 0.044*
	(0.028)	(0.013)	(0.014)	(0.006)	(0.006)	(0.013)
Pre-MSS trend	−0.003	0.005	−0.0028	− 0.008	0.008	0.004
	(0.008)	(0.008)	(0.006)	(0.004)	(0.007)	(0.008)
Observations	5163	5163	5404	5514	5514	5514
R-squared	0.116	0.164	0.226	0.166	0.188	0.357
Southern region						
Spillover effect	0.001	0.011	−0.014	0.018	0.002	0.008
	(0.053)	(0.030)	(0.019)	(0.031)	(0.018)	(0.016)
Pre-MSS trend	0.009	−0.002	−0.005	0.001	−0.001	0.005
	(0.012)	(0.009)	(0.007)	(0.010)	(0.003)	(0.009)
Observations	5497	5497	5970	6002	6002	6002
R-squared	0.133	0.145	0.120	0.092	0.145	0.181

Robust standard errors are in parentheses. *** *p* < 0.01, ** *p* < 0.05, * *p* < 0.1 Pre-MSS trend, pre-implementation trend (i.e. the trend in the indicated outcome prior to implementation of the midwives service scheme)

In the Northern region, there was a 5.4 percentage point increase (p-value = 0.011) in the use of delivery services at public sector clinics and a concurrent 4 percentage point decrease (p-value = 0.075) in home births in 2010. We found no evidence of any change in other outcomes in the Northern region. Similarly, we found no evidence of any change in the use of obstetric services among urban respondents in the south of the country.

## Discussion

We set out to gain more insight into mechanisms underlying reported MSS-induced changes in the antenatal clinic visits to rural public sector clinics and to determine if midwives’ attrition was associated with a spillover of the scheme’s effect into urban areas. In achieving the first goal, we obtained effect sizes that are identical to those in Okeke et al.’s (2016) study, [2, 3] and demonstrated that the reported increase in antenatal clinic visits to rural public sector clinics was driven by the program’s intended beneficiaries. Our results also suggest that women who sought care in private sector clinics (< 8% of women aged 15 – 50 years) were not attracted by the increased provider availability, reduced waiting time or potentially lower out of pocket cost of care at public sector clinics. [8] As previously demonstrated, the MSS-induced increase in antenatal clinic visits to rural public sector clinics was significant and larger in the northern region than the southern region. [1–3] Regarding our second goal, we demonstrated that in 2010, Northern urban public sector clinics witnessed no change in the use of antenatal services, but saw an increase in the use of delivery services. This increase was driven by women who would have otherwise delivered at home.

Nigeria has a critical shortage of midwives (and other skilled birth attendants) and those available usually provide care in urban clinics (see Table 1). [22, 23] This shortage limits coverage for after-hours care, and thus affects use of delivery care more than antenatal care since the latter usually occurs during regular business hours. [24–27] Midwives’ attrition reduced MSS clinics’ capacity to provide after-hours delivery care, and explains the level trend in use of delivery services in rural public sector clinics. [6] Midwives’ attrition also increased the availability of midwives in urban areas, which may have increased the capacity of urban (public sector) clinics to provide after-hours delivery care. This may explain why we observed a level trend in use of antenatal service, a 5-percentage point increase in use of delivery services in urban public sector clinics, and a 4 percentage point reduction in deliveries occurring away from clinics (see Table 5). There are other plausible explanations for our observations. For example, it is possible that most MSS facilities were in peri-urban areas. We are unable to verify this as data on the characteristics of MSS clinics, or their selection criteria are not publicly available. However, this explanation is unlikely because the program prioritized providing greater access to women in remote and hard to reach rural areas. A second plausible explanation is that there may have been one or more concurrent state-level demand inducing maternal interventions. In exploring this possibility, we found only one concurrent state-level intervention – the *Abiye* project in Ondo state – which was introduced between 2009 and 2010. [28–31] However, this explanation is also unlikely for two reasons: our results didn’t change when we excluded data from residents of Ondo and its neighboring states from our analyses; and Ondo state is in the South. A third plausible explanation is that the relatively faster rate of development and/or greater propensity for institutional delivery in urban areas (compared to rural areas) may have driven the increase in use of delivery services in urban clinics. However, this is unlikely for several reasons: the increase was restricted to urban public sector clinics; there was no change in antenatal clinic visits in urban clinics; when we re-centered respondents’ data around 2009 and 2008, we observed no change in outcomes of interest; and our conclusions were robust when we used alternative analytic methods (i.e. difference-in-difference and difference-in-discontinuities) that allowed us control for time (either linearly or using time fixed effects).

This study has some limitations. Although potentially useful, we did not provide estimates of the scheme’s ITTE and spillover effect by zone, because such analyses might be underpowered. We used a complete case analyses approach for two reasons: less than 4% of respondents’ data was missing; and we found no plausible explanation for the mechanism of missingness. We also did not assess for changes in the number of antenatal visits because between 2003 and 2013, per policy recommendation, many clinics may have switched from the traditional care model (requiring 10 – 13 antenatal visits on average) to the focused care model (requiring at least four antenatal visits on average in uncomplicated cases). [32–34] In this instance, the rate of adoption of the new model will constitute a time varying unobserved variable that would bias estimates. Lastly, some respondents that delivered in 2010 may have needed antenatal care in 2009. This could introduce measurement error and bias in our analyses. However, this is unlikely as our estimates closely match those from an earlier study. It is plausible that outcome trends in use of obstetric service are more amenable to non-linear specification. We explored this by specifying outcome trends using quadratic and cubic terms (with single trends as well as separate pre- and post trends in outcomes of interest), and we obtained similar results.

## Conclusion

MSS-induced increases in use of antenatal services at rural public-sector clinics were driven by the program’s intended beneficiaries. Additionally, midwife attrition from the MSS was associated with a spillover of the scheme’s effect on the use of delivery services in urban areas.

## Abbreviations

DHS: Demographic and Health Survey
ITS: Interrupted time series
ITTE: Intention-to-treat effect
MSS: Midwives Service Scheme
NDHS: Nigerian Demographic and Health Surveys
SURE–P MCH: Subsidy Reinvestment and Empowerment Program for Maternal and Child Health
